# Baseline Characteristics and Outcomes of Patients with Head and Neck Burn Injuries; a Cross-Sectional Study of 2181 Cases

**DOI:** 10.22037/aaem.v9i1.948

**Published:** 2020-12-11

**Authors:** Soudabeh Haddadi, Arman Parvizi, Reza Niknama, Shadman Nemati, Ramyar Farzan, Ehsan Kazemnejad

**Affiliations:** 1 Anesthesiology Research Center, Department of Anesthesiology, Alzahra Hospital, Guilan University of Medical Sciences, Rasht, Iran.; 2 Guilan University of Medical Sciences, Rasht, Iran.; 3 Otorhinolaryngology Research Center, Department of ENT, Head and Neck Surgery, Amiralmomenin Hospital, Guilan University of Medical Sciences, Rasht, Iran.; 4 Department of General Surgery, Velayat Hospital, Guilan University of Medical Sciences, Rasht, Iran.

**Keywords:** Burns, Patient outcome assessment, Intubation, intratracheal, Head, Neck, Respiration, artificial

## Abstract

**Introduction:**

Despite recent progress in treatment of burn injuries, head and neck burn and its complications is still considered a challenge. This study aimed to evaluate the baseline characteristics and outcomes of patients with head and neck burn.

**Methods:**

In this retrospective cross-sectional study, the medical profiles of patients with head and neck burn referring to a burn care center during 2 years were reviewed and analyzed regarding the baseline characteristics and outcomes of participants.

**Results:**

392 (17.97%) cases suffered from head and neck burns. The mean burn percentage of participants was 29.31 ± 24.78, and 126 (32.14%) cases required tracheal intubation. There was a direct correlation between length of hospital stay and the degree of burn (p < 0.001). The length of hospitalization for patients burned by electricity was longer than those burned by other mechanisms (p = 0.003). There was a significant correlation between degree of burn and abnormal laryngoscopy findings (p = 0.036), developing acute respiratory distress syndrome (ARDS) (p < 0.001) and pneumonia (p < 0.001), need for mechanical ventilation (p < 0.001), and mortality rate (p < 0.001).

**Conclusion:**

Based on the findings of the present study, the prevalence of head and neck burn injuries was about 18% and 32.14% of these cases required airway management. 19 (4.85%) cases developed ARDS, 41 (10.46%) developed pneumonia, and 50 (12.76%) cases died. There was a significant correlation between degree of burn and abnormal laryngoscopy findings, developing ARDS and pneumonia, need for mechanical ventilation, and mortality rate.

## Introduction

It is estimated that 180,000 people die from burn injuries every year worldwide. Non-fatal burns are among the leading causes of disability-adjusted life years (DALYs), which often occur in low- and middle-income countries. According to the World Health Organization (WHO), nearly 11 million people were severely burned worldwide in 2004, such that they required medical care (1). The proportion of head and neck burns in burn center admissions has been estimated as 47% in a study, indicating the importance of this type of injury (2).

Prevention of primary injury should always be a priority, but when the injury occurs, the primary goal should be to prevent the progression of injury and ensuring patient survival (3). Burns can cause extensive and devastating injury to the face and neck. Complications of burns can be divided into two categories: cosmetic and functional. Functional complications include airway injuries, carbon monoxide poisoning, corneal and eye burns, chondrites, and ear injuries (3-6). 

The most important part in the initial evaluation of patients after burns is to examine the airways and support them (3, 4). It has been estimated that approximately 13,000 to 22,000 people suffer from inhalation burns each year in the United States (4). Although the patient may be able to breathe spontaneously in the early hours after the burn, it is still possible for the injury to spread and affect the entire respiratory tract (3). Inhalation injury is the third leading predictor of death due to burn after total body surface area (TBSA) burn and aging. The mortality rate of burn victims with inhalation injuries has been estimated to be about 30%, while only 2% of burn patients without respiratory injury die (4).

Immediate primary care can have a significant impact on the rate of primary injury, its progression, and its long-term outcomes (2, 3). Inhalation burns have still remained a major challenge for anesthesiologists as well as intensive care and rehabilitation specialists worldwide, and there is no agreement on the appropriate and systematic evidence-based approach for treating the patients in the acute phase (4).

In order to plan for prevention and improving the level of care and outcomes of the head and neck burns, the first step is to assess the needs and outcomes of burn patients. Therefore, the present study aims to evaluate baseline characteristics and outcomes of patients with head and neck burns.

## Methods

***Study design and setting***

This retrospective, cross-sectional study was conducted on patients referring to Velayat Hospital (an academic burn care center in Rasht, North Iran), following face, head, neck, and airway burn injuries from April 2017 to the end of March 2019. Using the patients’ profiles, the baseline characteristics and outcomes of patients were collected and analyzed. The study protocol was approved by Vice President for Research of Guilan University of Medical Sciences (Ethics code: IR.GUMS.REC.1397.394). Researchers adhered to the principles of Helsinki Ethics recommendations and confidentiality of patients’ data.

Participants

All of the patients referring to Velayat Hospital, following face, head, and neck burn injuries from April 2017 to the end of March 2019 were included. Those who were discharged in less than 24 hours were excluded from the study. 

***Data gathering***

Using a predesigned checklist and census sampling, demographic characteristics, including age, gender, percentage of burn, etiology, burn scenario, comorbidity, first medical treatment, airway condition, need for mechanical ventilation, laryngoscopy finding, respiratory complications (acute respiratory distress syndrome (ARDS), pneumonia), length of hospital stay, and mortality rate were collected from the patients’ profiles. To determine the frequency of head and neck burns, all the records of head and neck burns and those unrelated to head and neck burns were counted in the specified time period. A medical student was responsible for data gathering. The percentage of burn was measured using rule of 9. 

The patients became candidates for intubation in case of drop in their saturation with suspected airway injury, presence of dyspnea, tachypnea, cyanosis, or loss of consciousness.

***Statistical analysis***

Data were analyzed using SPSS version 21.0 and presented as frequency (%) or mean ± standard deviation (SD). A p-value less than 0.05 was considered to be statistically significant.

## Results

***Baseline characteristics of participants***

Out of the 2181 patients referring to the studied hospital during 2017-2019, 392 (17.97%) cases suffered from head and neck burns. The mean age of patients was 37.14 ± 18.80 (0.5– 92) years (75.00% Male). Table 1 shows the baseline characteristics and outcomes of studied patients. The mean burn percentage of participants was 29.31 ± 24.78 (3 – 100) percent of body surface area (54.34% with 10-30% burns). Flame was the most common burn mechanism (52.3%) and the mean length of hospital stay was 6.02 ± 7.0 (1 -51) days. 

36.73% of females were burned by hot liquids, while the cause of burn in 58.16% of males was flame (p < 0.001). There were no statistically significant differences between male and female participants regarding the degree of burn (p = 0.109), airway condition (p = 0.861), laryngoscopic findings (p = 0.908), need for mechanical ventilation (p = 0.248), incidence of ARDS (p = 0.892), incidence of pneumonia (p = 0.775), and mortality (p = 0.861). 

***Outcomes***

126 (32.14%) cases required tracheal intubation. Laryngoscopic findings (in patients who required airway management) showed that 27 (21.43%) cases had erythema and edema. 19 (4.85%) cases developed ARDS, 41 (10.46%) developed pneumonia, and 50 (12.76%) cases died. There was a direct correlation between length of hospital stay and the degree of burn (p < 0.001). The length of hospitalization for patients who were burned by electricity was longer than those burned by other mechanisms (p = 0.003). 

Table 2 shows the correlation between laryngoscopic findings and baseline characteristics as well as outcomes of cases that underwent laryngoscopy. In addition, the correlation between mortality rate and baseline characteristics and outcomes of cases is shown in table 3. 

There was a significant correlation between degree of burn and abnormal laryngoscopy findings (p = 0.036), developing ARDS (p < 0.001) and pneumonia (p < 0.001), need for mechanical ventilation (p < 0.001), and mortality rate (p < 0.001). 

**Table1:** Baseline characteristics of patients who referred to the studied Hospital following head and neck burn injuries

**Variables**	**Number (%)**
**Gender**	
Female	98 (25.0)
Male	294 (75.0)
**Age (years)**	
> 20	58 (14.8)
20-40	170 (43.4)
40-60	116 (29.6)
≥ 60	48 (12.2)
**Mechanism of burn**	
Flame	205 (52.3)
Liquids	85 (21.7)
Electricity	13 (3.3)
Vapor	36 (9.2)
Chemicals	8 (2.0)
Unknown	45 (11.5)
**Outcomes**	
Need for tracheal intubation	126(32.14)
ARDS	19 (4.85)
Pneumonia	41 (10.46)
Mortality	50 (12.76)
Mean length of stay	6.02 ± 7.23
**Laryngoscopic view of candidate for intubation **	
Normal	99 (78.57)
Erythema, edema	27 (21.43)

Data are presented as mean ± standard deviation or frequency (%). ARDS: acute respiratory distress syndrome.

**Table 2:** Correlation between laryngoscopic findings and baseline characteristics as well as outcomes of cases who underwent laryngoscopy

**Variables**	**Laryngoscopic findings**		**P**
	**Normal (n= 99)**	Abnormal* (n = 27)	
**Burn degree **			
Second-degree	27 (27.27)	5 (18.52)	0.036
Second- to third	63 (63.63)	14 (51.85)	
Third- to fourth	9 (9.10)	8 (29.63)	
**Mechanism of burn**			
Flame	58 (58.59)	18 (66.67)	<0.001
Liquids	5 (5.05)	3 (11.11)	
Vapor	34 (34.34)	2 (7.41)	
Chemicals	1 (1.01)	0 (0.00)	
Unknown	1 (1.01)	4 (14.81)	
**Comorbidity**			
No	87 (87.88)	21 (77.78)	0.184
Yes	12 (12.12)	6 (22.22)	
**ARDS**			
No	92 (92.93)	16 (59.26)	<0.001
Yes	7 (7.07)	11 (40.74)	
**Pneumonia**			
No	83 (83.84)	9 (33.33)	<0.001
Yes	16 (16.16)	18 (66.67)	
**Mortality**			
No	72 (72.73)	8 (29.63)	<0.001
Yes	27 (27.27)	19 (70.37)	

Data are presented as frequency (%); Abnormal: erythema and edema. ARDS: acute respiratory distress syndrome.

**Table 3:** Correlation between mortality rate and baseline characteristics of cases with head and neck burn injuries

**Variables**	**Outcome**		**P**
	**Not survived (n = 50)**	**Survived (n = 342)**	
**Degree of burn**			
Second	6 (12.00)	183 (54.79)	<0.001*
Second- to third	36 (72.00)	143 (42.81)	
Third- to fourth	12 (24.00)	8 (2.40)	
Mechanism of burn			
Flame	35 (70.00)	170 (49.71)	0.121
Liquids	5 (10.00)	80 (23.39)	
Electricity	1 (2.00)	12 (3.51)	
Vapor	4 (8.00)	32 (9.36)	
Chemicals	0 (0.00)	8 (2.34)	
Unknown	5 (10.00)	40 (11.70)	
**Comorbidity**			
No	34 (68.00)	319 (93.27)	0.027
Yes	16 (32.00)	23 (6.73)	
**Tracheal intubation**			
No	16 (32.00)	326 (95.32)	<0.001
Yes	34 (68.00)	16 (4.68)	
**ARDS**			
No	37 (74.00)	336 (98.25)	<0.001
Yes	13 (26.00)	6 (1.75)	
**Pneumonia**			
No	29 (58.00)	322 (94.15)	<0.001
Yes	21 (42.00)	20 (5.85)	

Data are presented as number (%). ARDS: acute respiratory distress syndrome.

## Discussion

Based on the findings of the present study, the prevalence of head and neck burn injuries was about 18% and 32.14% of these cases required airway management. 19 (4.85%) cases developed ARDS, 41 (10.46%) developed pneumonia, and 50 (12.76%) cases died. There was a significant correlation between degree of burn and abnormal laryngoscopy findings, developing ARDS and pneumonia, need for mechanical ventilation, and mortality rate.

The prevalence of head and neck burn injuries in this study was much lower than that in the study by Hau Tian et al. (2020) who reported the percentage of head and neck burns among 1126 Chinese patients as 65.63% (7).

In the present study, 75% of the patients were male and 43.4% were in the age group of 20-40 years old, which was consistent with the report by the WHO (1). This result was also similar to the study by Hamilton et al. (2018) performed in the United States, in which 66% of the patients were male, however most of them were in their 40s (8). Also, in the study conducted by Hau Tian (2020) in China, 73.8% of the patients with head and neck burns were men (7), which was in line with the present study. This can be due to occupational exposure and workplace accidents. However, in another study conducted by Costa Santos (2016), women suffered from head and neck burns more than men, and the correlation between burn complications and gender was found to be statistically significant (9).

In their study, Burd et al. (2010) found that although patients may have spontaneous breathing in the first hours after a burn, there is always the possibility of spreading burn injury and edema throughout the respiratory tract (3), which can be caused by edema in the head and neck following the inhalation of vapor, smoke, or aspiration of burning liquids (3). 

In this study, only 32.14% of the patients needed intubation; 78.57% of them had normal glottis and 21.43% had erythema and edema. Also, 11.11% of the patients (N=3) with edematous and erythematous view in their laryngoscopy did not need intubation. This rate was significantly lower than the results of the study by Belba et al. (2008), who estimated the intubation rate for the patients with head and neck burns as 39% (10). The presence of soot in the mouth in facial and body burns necessitates a fiberoptic laryngoscopy, as it stabilizes the airways in the case of cutaneous lesions, inflammation, blisters, and significant wounds in these patients. The classic symptoms of inhalation injury, such as stridor, itching, shortness of breath, and dysplasia confirm the need for tracheal intubation (6).

The results of the present study showed that patients with second- and third-degree burns had a higher percentage of abnormal laryngoscopy. Also, patients with comorbidities had a higher rate of abnormal laryngoscopy (more than 3 times). Regarding the outcomes of burns based on laryngoscopy, it could be stated that intubation was observed in 88.89%, ventilation in 77.78%, ARDS in 40.74%, pneumonia in 66.67%, and mortality in 70.37% of the patients with abnormal laryngoscopy; these figures were significantly higher than those in the patients with normal laryngoscopy.

Costa Santos et al. (2016) found that head and neck burns significantly increased the incidence and severity of pneumonia in burn patients (9). In the present study, 10.46% of the patients developed pneumonia. Of the 392 patients with head and neck burns, 89.8% did not require mechanical ventilation (10.2% needed it) and ARDS was observed in 4.85% of the patients. However, in the study conducted by Miller et al. (2009), ARDS was identified as one of the leading causes of mortality, the incidence of which was estimated to be 20% (11).

In the study by Madnani et al. (2016), out of 40 patients in the emergency room, 8 patients required emergency intubation and their vocal cord edema was positive in laryngoscopy (P = 0.01)(12). In the present study, airway edema was also observed in vocal cords (13), and mortality rate following burns was 12.76%. Hamilton et al. (2018) in the United States found that although a quarter of patients with head and neck burns suffered from inhalation injuries, mortality occurred in only 2%. The researchers identified airway burns as an important risk factor for predicting mortality (8). The lower incidence of respiratory complications and higher mortality rate in the current study compared to other studies emphasizes the need for proper airway management and its vital role in the prognosis and survival of patients. In the present study, most of the patients had 10-30% burns, 48.95% of which were of the second-degree type. Flame was the most common type of burn mechanism among the patients with 52.3%. The mean length of hospital stay was 6.02±7 days. In the study by Hamilton et al. (2018), the mean length of hospital stay was 4.4 days and, as in the present study, flame was the most common cause of burns, which occurred locally and superficially (8). In another study, Bai et al. (2013) stated that ventilator support was significantly associated with increased length of hospitalization (13). However, in the study by Santos et al. (2016), those who were intubated were discharged earlier (9), which may be due to the high incidence of primary mortality in this group. In the present study, the length of hospital stay correlated with degree of burn, burn mechanism, airway condition, intubation, and incidence of ADRS and pneumonia. 

Inhalation injuries in patients with head and neck burns are serious and life-threatening, and the management of airways and respiratory outcomes over the hospitalization course of these patients should be taken into consideration.

## Limitations:

One of the most important limitations was the presence of incomplete patient profiles, which was due to the retrospective fashion of the study.

## Conclusion:

Based on the findings of the present study, the prevalence of head and neck burn injuries was about 18% and 32.14% of these cases required airway management. 19 (4.85%) cases developed ARDS, 41 (10.46%) developed pneumonia, and 50 (12.76%) cases died. There was a significant correlation between degree of burn and abnormal laryngoscopy findings, developing ARDS and pneumonia, need for mechanical ventilation, and mortality rate.
